# Etymologia: Parvovirus

**DOI:** 10.3201/eid2402.ET2402

**Published:** 2018-02

**Authors:** Eduardo K.U.N. Fonseca

**Keywords:** etymologia, parvovirus, parvovirus B19, viruses

## Parvovirus [pahr′ vo-vi′′res]

Viruses of the family *Parvoviridae* (Latin *parvum* [meaning small or tiny]) are among the smallest viruses described, 18–28 nm in diameter (Figure). There are 2 subfamilies of the family *Parvoviridae*: *Parvovirinae* and *Densovirina* (Latin *denso* [thick or compact]). *Parvovirinae* may infect humans, but *Densovirina* infect only arthropods (*1*). Structurally, these viruses are nonenveloped, icosahedral viruses that contain a single-stranded linear DNA genome (*2*,*3*).

**Figure:**
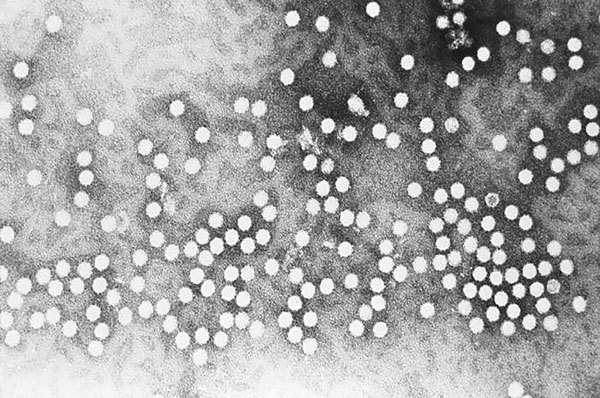
This electron micrograph depicts a number of parvovirus H-1 virions of the Parvoviridae family of DNA viruses. Photo CDC/ R. Regnery; E. L. Palmer, 1981.

The small size of these viruses might account for their late discovery. In 1974, the first pathogenic human parvovirus was discovered and named B19 from the coding of a serum sample, number 19 in panel B, that gave anomalous results during testing for hepatitis B (*4*). Although human B19 infections are more often asymptomatic or lead to mild rash illnesses and arthralgias, they can also cause severe anemia in fetuses and in persons with underlying hemoglobinopathies (*5*).
